# Comparison of Outcomes in Elective Endovascular Aortic Repair vs Open Surgical Repair of Abdominal Aortic Aneurysms

**DOI:** 10.1001/jamanetworkopen.2019.6578

**Published:** 2019-07-10

**Authors:** Konrad Salata, Mohamad A. Hussain, Charles de Mestral, Elisa Greco, Badr A. Aljabri, Muhammad Mamdani, Thomas L. Forbes, Deepak L. Bhatt, Subodh Verma, Mohammed Al-Omran

**Affiliations:** 1 Division of Vascular Surgery, University of Toronto, Toronto, Ontario, Canada; 2 Division of Vascular Surgery, Li Ka Shing Knowledge Institute, St Michael’s Hospital, Toronto, Ontario, Canada; 3 Department of Surgery, King Saud University, Riyadh, Kingdom of Saudi Arabia; 4 Li Ka Shing Centre for Healthcare Analytics Research and Training (CHART), Li Ka Shing Knowledge Institute, St Michael’s Hospital, Toronto, Ontario, Canada; 5 Leslie Dan Faculty of Pharmacy, University of Toronto, Toronto, Ontario, Canada; 6 Division of Vascular Surgery, Peter Munk Cardiac Centre, University Health Network, Toronto, Ontario, Canada; 7 Brigham and Women’s Hospital Heart and Vascular Center, Boston, Massachusetts; 8 Harvard Medical School, Boston, Massachusetts; 9 Division of Cardiac Surgery, Li Ka Shing Knowledge Institute, St Michael’s Hospital, Toronto, Ontario, Canada; 10 Division of Vascular Surgery, University of Toronto, Toronto, Ontario, Canada

## Abstract

**Importance:**

Knowledge regarding the long-term outcomes of elective treatment of abdominal aortic aneurysm (AAA) using endovascular aortic repair (EVAR) is increasing. However, data with greater than 10 years’ follow-up remain sparse and are lacking from population-based studies.

**Objective:**

To determine the long-term outcomes of EVAR compared with open surgical repair (OSR) for elective treatment of AAA.

**Design, Setting, and Participants:**

This retrospective, population-based cohort study used linked administrative health data from Ontario, Canada, to identify all patients 40 years and older who underwent elective EVAR or OSR for AAA repair from April 1, 2003, to March 31, 2016, with follow-up terminating on March 31, 2017. A total of 17 683 patients were identified using validated procedure and billing codes and were propensity score matched. Analysis was conducted from June 26, 2018, to January 16, 2019.

**Exposures:**

Elective EVAR or OSR for AAA.

**Main Outcomes and Measures:**

The primary outcome was overall survival. Secondary outcomes were major adverse cardiovascular event–free survival, defined as being free of death, myocardial infarction, or stroke; reintervention; and secondary rupture.

**Results:**

Among 17 683 patients who received elective AAA repairs (mean [SD] age, 72.6 [7.8] years; 14 286 [80.8%] men), 6100 (34.5%) underwent EVAR and 11 583 (65.5%) underwent OSR. From these patients, 4010 well-balanced propensity score–matched pairs of patients were defined, with a mean (SD) age of 73.0 (7.6) years and 6583 (82.1%) men. In the matched cohort, the mean (SD) follow-up was 4.4 (2.7) years, and maximum follow-up was 13.8 years. The overall median survival was 8.9 years. Compared with OSR, EVAR was associated with a higher survival rate up to 1 year after repair (91.0% [95% CI, 90.1%-91.9%] vs 94.0% [95% CI, 93.3%-94.7%]) and a higher major adverse cardiovascular event–free survival rate up to 4 years after repair (69.9% [95% CI, 68.3%-71.3%] vs 72.9% [95% CI, 71.4%-74.4%]). Cumulative incidence of reintervention was higher among patients who underwent EVAR compared with those who underwent OSR at the 7-year follow-up (45.9% [95% CI, 44.1%-47.8%] vs 42.2% [95% CI, 40.4%-44.0%]). Survival analyses demonstrated no statistically significant differences in long-term survival, reintervention, and secondary rupture for patients who underwent EVAR compared with those who underwent OSR. Kaplan-Meier analysis suggested superior long-term major adverse cardiovascular event–free survival among patients who underwent EVAR compared with those who underwent OSR (32.6% [95% CI, 26.9%-38.4%] vs 14.1% [95% CI, 4.0%-30.4%]; stratified log-rank *P* < .001) during a maximum follow-up of 13.8 years.

**Conclusions and Relevance:**

Endovascular aortic repair was not associated with a difference in long-term survival during more than 13 years’ maximum follow-up. The reasons for these findings will require studies to consider specific graft makes and models, adherence to instructions for use, and types and reasons for reintervention.

## Key Points

**Question:**

What are the long-term outcomes of elective endovascular aortic repair of abdominal aortic aneurysms compared with those of open surgical repair?

**Findings:**

In this population-based cohort study including 17 683 patients receiving elective treatment for abdominal aortic aneurysms, no statistically significant difference was found in long-term all-cause mortality between endovascular aortic repair and open surgical repair during a maximum follow-up of 13.8 years.

**Meaning:**

Endovascular aortic repair was not associated with a long-term survival benefit.

## Teaser

This population-based cohort study compares long-term outcomes of elective endovascular aortic repair vs open surgical repair of abdominal aortic aneurysm among patients 40 years and older in Ontario, Canada.

## Introduction

Endovascular aortic repair (EVAR) has changed the landscape of abdominal aortic aneurysm (AAA) treatment since its introduction in 1991.^1^ Randomized and population-based studies investigating EVAR vs open surgical repair (OSR) demonstrated superior perioperative survival as well as significant improvements in operative time, blood loss, transfusion requirements, cardiopulmonary complications, and reduced lengths of stay in intensive care units and hospitals in favor of EVAR.^2,3,4^ Consequently, EVAR has seen rapid uptake and has become the predominant approach to AAA management, as borne out by multiple Canadian,^5^ US,^6,7^ and European^8^ studies.

Despite its clear short-term superiority, early EVAR technology had unique complications, including endoleak, graft migration, graft thrombosis, and secondary rupture.^9,10,11,12^ Accordingly, patients who undergo EVAR require lifelong surveillance to identify and prevent these complications, and EVAR is associated with significantly higher reintervention rates.^13^ These are at least in part postulated to be the reasons for the eventual loss of the EVAR mortality benefit demonstrated in EVAR randomized clinical trials (RCTs).^14,15,16,17^ However, since the inception of these trials in the 1990s and early 2000s, EVAR endografts have undergone numerous manufacturing reiterations to address identified endograft-related complications. As a result, the longevity of the mortality benefit of contemporary EVAR may be underestimated by these trials. On the other hand, limited data are available to elucidate whether EVAR is safe in the long term. Presently, the Endovascular Aortic Repair 1 (EVAR-1) trial^17^ and Dutch Randomized Endovascular Aneurysm Management (DREAM)^18^ trial are the only trials to report longer than 10-year follow-up, and no population-based studies have been conducted with longer than 10 years of follow-up, to our knowledge.

This study aims to add to the limited literature regarding the long-term outcomes of EVAR. Specifically, the objective of this study is to assess the differences between EVAR and OSR for elective AAA repair in long-term survival, major adverse cardiovascular event (MACE)–free survival, reintervention, and secondary rupture. This developing body of knowledge will serve to better delineate the true mortality benefit of EVAR, considering accumulated experience, evolved endografts, and better knowledge of complications and their appropriate management.

## Methods

### Study Design, Setting, and Data Sources

The population of Ontario, Canada, is 14.2 million people. A retrospective, population-based cohort study of EVAR vs OSR for elective AAA management in Ontario, Canada, was performed using administrative health care data, in accordance with the Strengthening the Reporting of Observational Studies in Epidemiology (STROBE) reporting guideline for cohort studies. The Ontario Ministry of Health and Long-Term Care records each publicly insured ambulatory, emergency, and inpatient health care system interaction requiring the use of an Ontario health card. The Institute for Clinical Evaluative Sciences, a prescribed entity governed under the Personal Health Information Protection Act, stores and manages these data. Data are linked together across primary sources using a unique encrypted identifier known as the Institute for Clinical Evaluative Sciences key number. Ontario administrative data are routinely used for population-level research and have previously been thoroughly described and validated.^19,20,21,22^ The specific databases used for this study are described in eTable 1 in the Supplement. This study was approved by the research ethics board at Sunnybrook Health Sciences Centre in Toronto, Ontario, Canada, and the requirement for informed consent was waived owing to the use of deidentified secondary data.

### Patient Cohort

All elective EVARs and OSRs of AAA performed in Ontario, Canada, in patients 40 years and older from April 1, 2003, to March 31, 2016, with maximum follow-up terminating on March 31, 2017, were identified. This age cutoff was used to reduce the likelihood of contamination with connective tissue aneurysms. Combinations of the *International Statistical Classification of Diseases and Related Health Problems, Tenth Revision, Canadian Revision*; *Canadian Classification of Health Intervention*; and Ontario Health Insurance Plan diagnostic, procedure, and billing codes were used according to a previously validated algorithm.^22^ This algorithm identified EVAR (infrarenal) and OSR (infrarenal, pararenal, and juxtarenal) with 96% and 95% positive predictive values, respectively. Patients with multiple AAA repair procedures listed on their index admission were excluded owing to an inability to establish appropriate event chronology and, thus, the primary procedure. Patients who relocated outside of Ontario or whose health cards expired prior to study conclusion were also excluded.

### Covariates

Baseline covariates included demographic characteristics, health care utilization, hospital and procedure characteristics, comorbidities, medications, and tracer variables (used to help demonstrate absence of residual confounding). A full list of covariates, definitions, corresponding codes, and references to respective validation studies is presented in eTable 2 in the Supplement. All baseline covariates were captured using a 5-year lookback window from the index procedure date, except those captured from the National Ambulatory Care Reporting System database, where a 3-year lookback window was used, as this database commenced in July 2000. All medication variables, except for fluoroquinolones, were measured using a 4-month lookback window to assess whether at least 1 prescription was recently filled, considering a 100-day maximum medication dispensing limit. In the province of Ontario, patients younger than 65 years are not eligible for publicly funded pharmaceutical care; as such, drug information is not captured for these individuals within administrative data. Patients younger than 65 years without drug information were coded as ineligible for the Ontario Drug Benefit program.

### Outcomes and Follow-up

The primary outcome was overall survival. Secondary outcomes included MACE-free survival, defined as survival free of a composite of death, stroke, or myocardial infarction; any reintervention; and secondary rupture. These outcomes were defined according to previously validated codes where possible (eTable 2 in the Supplement). Patients were observed through administrative databases until the outcome of interest or end of the follow-up period on March 31, 2017, whichever happened first.

### Statistical Analysis

A propensity score–matched survival analysis was performed.^23,24^ The propensity score for repair approach was calculated using a logistic regression model incorporating all covariates as potential confounders. Patients who received EVAR or OSR were matched 1:1 using the greedy nearest neighbor method with a caliper width of 0.2 SD units. Balance of covariates was assessed using standardized differences, with differences less than 0.1 indicating good balance.^25^ Residual confounding was assessed using the distribution of tracer variables not used to specify the propensity score. Survival analyses were conducted using the Kaplan-Meier product limit method to assess differences in survival and MACE-free survival. Statistical differences were assessed using the stratified log-rank test with stratification on the matched pairs. To account for competing risks, cumulative incidence function analysis using a Fine and Gray model and stratified Gray test were conducted to assess differences in reintervention and secondary rupture. Statistical significance was set at a Bonferroni-corrected *P* value of 1.25 × 10^−5^ to adjust for the 4010 matched-pair comparisons.^26,27^ Survival and cumulative incidence rates and associated 95% CIs were obtained from corresponding survival and cumulative incidence functions at 30 days, annually for up to 10 years, as well as at maximum follow-up.

The calculated propensity score was used to conduct an inverse probability of treatment–weighted sensitivity analysis.^28^ The mean treatment effect in the treated weight was used, and balance of covariates was assessed using standardized mean differences as well as variance ratios, with ratio values between 0.5 and 2.0 indicating good balance.^29,30^ Cox regression models with a robust sandwich variance estimator were then used to calculate hazard ratios (HRs) for each of the outcomes.^31^ Cause-specific Cox models were fit for the reintervention and secondary rupture outcomes to account for competing risks. Proportionality of hazards was investigated using log-log survival plots and time-dependent covariates. Where hazards were not proportional, data were partitioned into multiple time intervals, and separate Cox models were built for each interval. Cutoff values were determined using plots of Schoenfeld residuals through time and tested as previously described. Statistical significance was set at a 2-tailed *P* value less than .05. All statistical analyses were conducted in SAS statistical software version 9.4 (SAS Institute). Analysis was conducted from June 26, 2018, to January 16, 2019.

## Results

### Cohort Characteristics

The overall study population consisted of 17 683 patients who received elective AAA repairs (mean [SD] age, 72.6 [7.8] years; 14 286 [80.8%] men). Of these patients, 6100 (34.5%) underwent EVAR and 11 583 (65.5%) underwent OSR (Table). In the overall cohort, patients who underwent EVAR were more likely to be older, be men, reside in areas with populations more than 10 000 people, and use the health care system more frequently. Furthermore, patients who underwent EVAR were more likely to have comorbid congestive heart failure, diabetes, hypertension, and a Charlson Comorbidity Index score of 2 or more, while patients who underwent OSR were more likely to have underlying peripheral arterial disease. Patients older than 65 years who underwent EVAR were more likely to be prescribed statin, angiotensin-converting enzyme inhibitor or angiotensin receptor blocker, anticoagulant, or noninsulin antidiabetes medications or prednisone and were more likely to have a history of fluoroquinolone use. Additionally, EVAR procedures within the unmatched cohort were more commonly conducted in teaching hospitals or high-volume hospitals (defined as ≥10 AAA procedures per year) and were more common than OSR after 2009. The propensity matched–cohort included 4010 pairs of patients (mean [SD] age, 73.0 [7.6] years; 6583 [82.1%] men) who underwent EVAR or OSR and did not demonstrate any significant differences in baseline covariates or tracer variables.

**Table.** Baseline Characteristics of Unmatched and Propensity Score–Matched Cohorts

Variable	Unmatched Cohort			Matched Cohort		
	No. (%)		Standardized Difference	No. (%)		Standardized Difference
	EVAR (n = 6100)	OSR (n = 11 583)		EVAR (n = 4010)	OSR (n = 4010)	
**Demographic Characteristics**						
Age, mean (SD), y	75.3 (7.9)	71.2 (7.8)	0.52	73.1 (7.7)	72.8 (7.4)	0.03
Men	5159 (84.6)	9127 (78.8)	0.15	3310 (82.5)	3273 (81.6)	0.02
Income quintile						
1 (Lowest)	1135 (18.6)	2342 (20.2)	0.04	753 (18.8)	758 (18.9)	0
2	1304 (21.4)	2517 (21.7)	0.01	849 (21.2)	851 (21.2)	0
3	1166 (19.1)	2276 (19.6)	0.01	803 (20.0)	788 (19.7)	0.01
4	1260 (20.7)	2306 (19.9)	0.02	823 (20.5)	814 (20.3)	0.01
5 (Highest)	1226 (20.1)	2102 (18.1)	0.05	774 (19.3)	790 (19.7)	0.01
Not reported	9 (0.1)	40 (0.3)	0.04	8 (0.2)	9 (0.2)	0.01
Rural residence	922 (15.1)	2244 (19.4)	0.11	691 (17.2)	700 (17.5)	0.01
Health care utilization, mean (SD), No.						
Outpatient physician visits within 1 y	14.4 (8.2)	13.4 (7.5)	0.12	13.6 (7.5)	13.4 (7.7)	0.02
Emergency department visits within 3 y	2.4 (3.8)	1.9 (3.0)	0.14	2.1 (3.5)	2.1 (3.4)	0.01
Hospital admissions within 3 y	0.8 (1.3)	0.7 (1.1)	0.15	0.7 (1.2)	0.7 (1.2)	0.02
**Comorbidities**						
Charlson Comorbidity Index score						
0	902 (14.8)	1592 (13.7)	0.03	571 (14.2)	579 (14.4)	0.01
1	916 (15.0)	1846 (15.9)	0.03	607 (15.1)	627 (15.6)	0.01
≥2	1605 (26.3)	2104 (18.2)	0.20	876 (21.8)	856 (21.3)	0.01
Not reported	2677 (43.9)	6041 (52.2)	0.17	1956 (48.8)	1948 (48.6)	0
Coronary artery disease	1082 (17.7)	1957 (16.9)	0.02	672 (16.8)	668 (16.7)	0
Myocardial infarction	373 (6.1)	640 (5.5)	0.03	223 (5.6)	219 (5.5)	0
Congestive heart failure	1082 (17.7)	1183 (10.2)	0.22	527 (13.1)	497 (12.4)	0.02
Peripheral artery disease	169 (2.8)	1567 (13.5)	0.40	131 (3.3)	132 (3.3)	0
Cerebrovascular disease	78 (1.3)	191 (1.6)	0.03	61 (1.5)	60 (1.5)	0
Stroke or transient ischemic attack	226 (3.7)	401 (3.5)	0.01	131 (3.3)	141 (3.5)	0.01
Diabetes	1877 (30.8)	2711 (23.4)	0.17	1172 (29.2)	1148 (28.6)	0.01
Hypertension	5025 (82.4)	8944 (77.2)	0.13	3243 (80.9)	3228 (80.5)	0.01
Chronic obstructive pulmonary disease	2530 (41.5)	4247 (36.7)	0.10	1614 (40.2)	1575 (39.3)	0.02
Chronic kidney disease	161 (2.6)	197 (1.7)	0.06	89 (2.2)	71 (1.8)	0.03
Prior procedure						
Coronary revascularization	700 (11.5)	1234 (10.7)	0.03	461 (11.5)	453 (11.3)	0.01
Peripheral revascularization	1276 (20.9)	2694 (23.3)	0.06	815 (20.3)	841 (21.0)	0.02
Major amputation	1-5^a^	14 (0.1)	0.03	1-5^a^	1-5^a^	0.03
Carotid revascularization	72 (1.2)	192 (1.7)	0.04	57 (1.4)	55 (1.4)	0
Medication						
Statins	3712 (60.9)	5889 (50.8)	0.20	2336 (58.3)	2317 (57.8)	0.01
β-Blockers	2208 (36.2)	3903 (33.7)	0.05	1359 (33.9)	1333 (33.2)	0.01
Angiotensin-converting enzyme inhibitors or angiotensin receptor blockers	3279 (53.8)	5355 (46.2)	0.15	2046 (51.0)	2024 (50.5)	0.01
Antiplatelets	6 (0.1)	31 (0.3)	0.04	6 (0.1)	<6^a^	0.01
Anticoagulants	750 (12.3)	696 (6.0)	0.22	353 (8.8)	336 (8.4)	0.02
Fluoroquinolones	543 (8.9)	703 (6.1)	0.11	303 (7.6)	289 (7.2)	0.01
Antidiabetics	774 (12.7)	1012 (8.7)	0.13	485 (12.1)	468 (11.7)	0.01
Prednisone	328 (5.4)	336 (2.9)	0.12	166 (4.1)	162 (4.0)	0.01
Insulin	122 (2.0)	112 (1.0)	0.09	72 (1.8)	66 (1.6)	0.01
Ontario drug benefit–eligible	5469 (89.7)	9196 (79.4)	0.29	3432 (85.6)	3390 (84.5)	0.03
**Procedure and Institution Characteristics**						
Hospital type						
Teaching	4966 (81.4)	6442 (55.6)	0.58	3008 (75.0)	2993 (74.6)	0.01
High volume	5563 (91.2)	10 205 (88.1)	0.10	3 626 (90.4)	3 615 (90.1)	0.01
Year of procedure						
2003	18 (0.3)	954 (8.2)	0.40	18 (0.4)	15 (0.4)	0.01
2004	12 (0.2)	1321 (11.4)	0.49	12 (0.3)	10 (0.2)	0.01
2005	13 (0.2)	1374 (11.9)	0.50	13 (0.3)	9 (0.2)	0.02
2006	147 (2.4)	1114 (9.6)	0.31	144 (3.6)	143 (3.6)	0
2007	289 (4.7)	996 (8.6)	0.16	269 (6.7)	275 (6.9)	0.01
2008	409 (6.7)	878 (7.6)	0.03	345 (8.6)	352 (8.8)	0.01
2009	538 (8.8)	847 (7.3)	0.06	425 (10.6)	413 (10.3)	0.01
2010	575 (9.4)	756 (6.5)	0.11	410 (10.2)	408 (10.2)	0
2011	714 (11.7)	758 (6.5)	0.18	469 (11.7)	492 (12.3)	0.02
2012	784 (12.9)	635 (5.5)	0.26	444 (11.1)	469 (11.7)	0.02
2013	779 (12.8)	589 (5.1)	0.27	435 (10.8)	424 (10.6)	0.01
2014	777 (12.7)	639 (5.5)	0.25	471 (11.7)	452 (11.3)	0.01
2015	805 (13.2)	580 (5.0)	0.29	440 (11.0)	428 (10.7)	0.01
2016	240 (3.9)	142 (1.2)	0.17	115 (2.9)	120 (3.0)	0.01
Tracer variables						
Cataract surgery	1196 (19.6)	1796 (15.5)	0.11	690 (17.2)	740 (18.5)	0.03
Malignancy	1152 (18.9)	1548 (13.4)	0.15	665 (16.6)	597 (14.9)	0.05

Abbreviations: EVAR, endovascular aortic repair; OSR, open surgical repair.

^a^ Cell value is represented as a range to eliminate patient reidentification risk, according to mandatory Institute for Clinical Evaluative Sciences practice.

### Outcomes

Mean (SD) follow-up for the entire cohort was 5.5 (3.6) years with a maximum follow-up of 14.0 years. Among the matched cohort, mean (SD) follow-up was 4.4 (2.7) years with a maximum follow-up of 13.8 years. Median survival for the unmatched cohort was 9.4 years overall, 7.8 years among patients who underwent EVAR, and 9.8 years among patients who underwent OSR. In the matched cohort, median survival was 8.9 years overall and in each treatment group.

Within the matched cohort, EVAR was associated with a higher survival rate than OSR for up to 1 year after repair (94.0% [95% CI, 93.3%-94.7%] vs 91.0% [95% CI, 90.1%-91.9%]), and a higher MACE-free survival rate for up to 4 years after repair (72.9% [95% CI, 71.4%-74.4%] vs 69.9% [95% CI, 68.3%-71.3%]) (eTable 3 in the Supplement). The cumulative incidence of reintervention within 30 days was lower among patients who underwent EVAR compared with OSR (12.4% [95% CI, 11.4%-13.5%] vs 15.0% [95% CI, 13.9%-16.1%]) but higher after 7 years (45.9% [95% CI, 44.1%-47.8%] vs 42.2% [95% CI, 40.4%-44.0%]) (eTable 3 in the Supplement). No significant differences in the cumulative incidence of secondary rupture were demonstrated within the matched cohort at maximum follow-up (EVAR, 1.5% [95% CI, 0.9%-2.5%]; OSR, 0.8% [95% CI, 0.6%-1.2%]) (eTable 3 in the Supplement).

**Figure 1.**
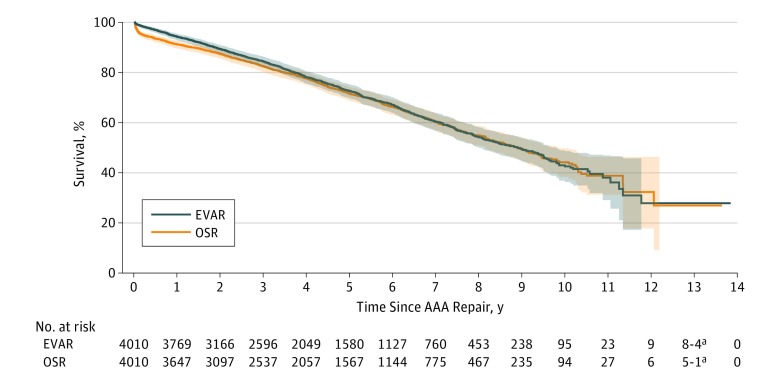
Kaplan-Meier Curve of Survival After Elective Abdominal Aortic Aneurysm (AAA) Treatment by Endovascular Aortic Repair (EVAR) and Open Surgical Repair (OSR) Stratified log-rank *P* = .004. Bonferroni-corrected significance at *P* < 1.25 × 10^−5^. Shading indicates 95% confidence bands. ^a^Number at risk value or difference between adjacent values is less than 6. Value is represented as a range to eliminate patient reidentification risk, according to mandatory Institute for Clinical Evaluative Sciences practice.

**Figure 2.**
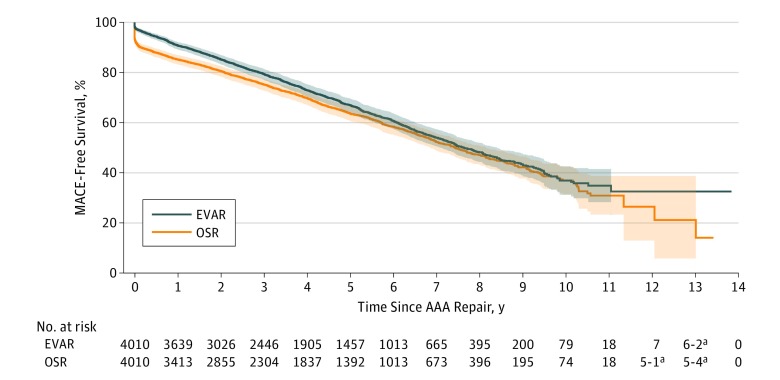
Kaplan-Meier Curve of Major Adverse Cardiovascular Event (MACE)–Free Survival After Elective Abdominal Aortic Aneurysm (AAA) Treatment by Endovascular Aortic Repair (EVAR) and Open Surgical Repair (OSR) Stratified log-rank *P* = 1.06 × 10^−7^. Bonferroni-corrected significance set at *P* < 1.25 × 10^−5^. Shading indicates 95% confidence bands. ^a^Number at risk value or difference between adjacent values is less than 6. Value is represented as a range to eliminate patient re-identification risk, according to mandatory Institute for Clinical Evaluative Sciences practice.

**Figure 3.**
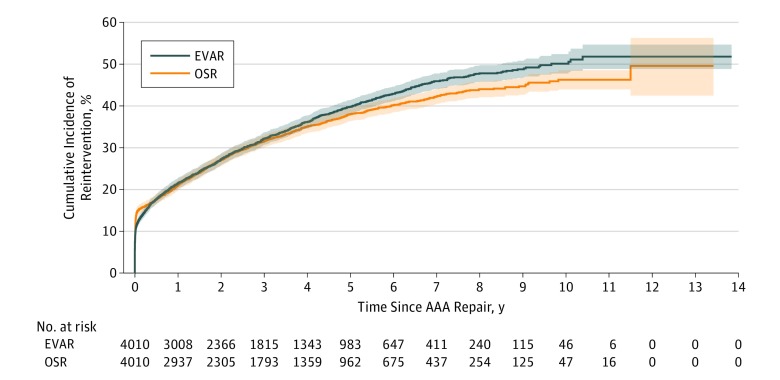
Cumulative Incidence Function Curve for Reintervention Following Elective Abdominal Aortic Aneurysm (AAA) Treatment by Endovascular Aortic Repair (EVAR) and Open Surgical Repair (OSR) Stratified Gray test *P* = .68. Bonferroni-corrected significance set at *P* < 1.25 × 10^−5^. Shading indicates 95% confidence bands.

**Figure 4.**
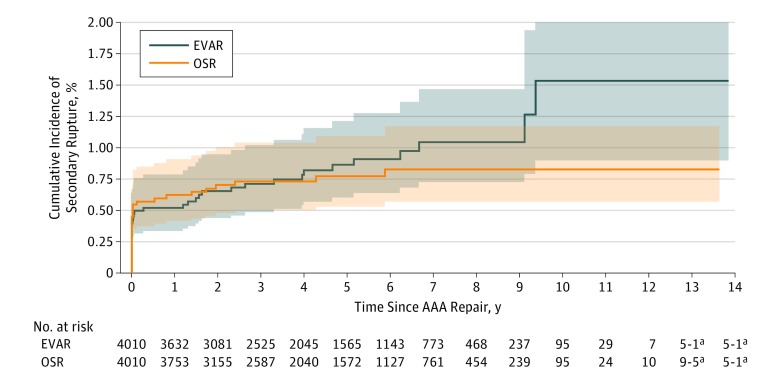
Cumulative Incidence Function Curve for Secondary Rupture Following Elective Abdominal Aortic Aneurysm (AAA) Treatment by Endovascular Aortic Repair (EVAR) and Open Surgical Repair (OSR) Stratified Gray test *P* = .69. Bonferroni-corrected significance set at *P* < 1.25 × 10^−5^. Shading indicates 95% confidence bands. ^a^Number at risk value or difference between adjacent values is less than 6. Value is represented as a range to eliminate patient reidentification risk, according to mandatory Institute for Clinical Evaluative Sciences practice.

Kaplan-Meier survival analysis demonstrated no statistically significant differences between EVAR and OSR in long-term survival (at maximum follow-up: EVAR, 41.5% [95% CI, 37.7%-45.2%]; OSR, 26.9% [95% CI, 15.7%-39.5%]; stratified log-rank *P* = .004) (Figure 1). However, EVAR was associated with superior long-term MACE-free survival (at maximum follow-up: EVAR, 32.6% [95% CI, 26.9%-38.4%]; OSR, 14.1% [95% CI, 4.0%-30.4%]; stratified log-rank *P* = 1.06 × 10^−7^) (Figure 2). No statistically significant differences in long-term reintervention (at maximum follow-up: EVAR, 51.8% [95% CI, 48.8%-54.7%]; OSR, 49.6% [95% CI, 42.5%-56.3%]; stratified Gray test *P* = .68) (Figure 3) or secondary rupture (at maximum follow-up: EVAR, 1.5% [95% CI, 0.9%-2.5%]; OSR, 0.8% [95% CI, 0.6%-1.2%]; stratified Gray test, *P* = .69) were noted (Figure 4).

### Sensitivity Analysis

The inverse probability of treatment–weighted cohort was balanced on all covariates. Cox models demonstrated similar findings between the propensity score–matched cohort and inverse probability of treatment–weighted cohort (eTable 4 in the Supplement). Endovascular aortic repair was associated with lower risk of all-cause mortality within 45 days of the procedure (HR, 0.29; 95% CI, 0.22-0.40; *P* < .001). However, between 1 to 4 years’ follow-up, EVAR was associated with higher all-cause mortality (HR, 1.16; 95% CI, 1.02-1.32; *P* = .02). Similarly, EVAR was associated with a lower risk of MACE within 45 days of the procedure (HR, 0.32; 95% CI, 0.27-0.39; *P* < .001) but no subsequent differences outside of the perioperative period. The risk of reintervention within 30 days of repair was significantly lower among patients who underwent EVAR (HR, 0.74; 95% CI, 0.66-0.83; *P* < .001) but significantly higher from 30 days to 6 months (HR, 1.36; 95% CI, 1.12-1.65; *P* = .002) and after 2 years (HR, 1.11; 95% CI, 1.01-1.22; *P* = .03) (eTable 3 in the Supplement). There were no statistically significant differences in secondary rupture among the matched or sensitivity cohorts.

## Discussion

This population-based, retrospective cohort study involving 4010 pairs of propensity score–matched patients who underwent EVAR or OSR observed for longer than 13 years demonstrated no statistically significant differences in long-term survival, reintervention, or secondary rupture between patients who underwent EVAR vs those who underwent OSR. In contrast, EVAR was associated with higher MACE-free survival throughout follow-up.

The results of this study complement the limited existing long-term data comparing EVAR with OSR for elective AAA repair. To our knowledge, the longest reported follow-up of patients who underwent EVAR compared with those who underwent OSR to date comes from the EVAR-1 multicenter RCT^17^ of 1252 patients observed for a mean (SD) of 12.7 (1.5) years. That trial demonstrated superior all-cause mortality among patients who underwent EVAR for up to 6 months following repair, but this mortality benefit was lost after 6 months, and patients who underwent EVAR demonstrated a higher hazard for all-cause mortality after 8 years (adjusted HR [aHR], 1.25; 95% CI, 1.00-1.56; *P* = .048).^17^ Similarly, the 9-year results of the Standard Open Surgery Versus Endovascular Repair of AAA (OVER) RCT^16^ showed loss of the EVAR mortality benefit by 3 years (HR, 0.72; 95% CI, 0.51-1.00; *P* = .05). Additionally, in the longest and largest population-based follow-up of patients who underwent EVAR or OSR, to our knowledge, Schermerhorn et al^32^ demonstrated lower short-term mortality for patients who underwent EVAR within 30 days (aHR, 0.32; 95% CI, 0.29-0.35; *P* < .001) and from 30 to 90 days of repair (aHR, 0.64; 95% CI, 0.58-0.71; *P* < .001), with worse outcomes at up to 4 years (aHR, 1.17; 95% CI, 1.13-1.21; *P* < .001) and after 4 years (aHR, 1.05; 95% CI; 1.00-1.09; *P* = .03). These latter results were most congruent with the findings of our study.

With the accumulation of long-term data, it is becoming clearer that the superiority of EVAR may be limited to the short term. One prevalent explanation for this finding is the high reintervention rate associated with EVAR. The EVAR-1 trial^17^ reported a 15-year reintervention rate of 26% in the EVAR group and an aHR of 6.29 (95% CI, 3.09-12.78; *P* < .001) for any reintervention between 6 months and 4 years after EVAR, while the DREAM trial^14^ reported a reintervention rate of approximately 30% at 6 years follow-up, and the OVER trial^16^ reported a reintervention rate of approximately 20% at 9 years follow-up. However, the seminal EVAR vs OSR RCTs used considerable numbers of now obsolete grafts (approximately 50%). It follows, then, that contemporary, well-adjusted population-based studies of newer grafts with accumulated technical experience should demonstrate a prolongation of the EVAR mortality benefit and a reduction of reintervention rates. This was not the case in our study, as reintervention rates mirrored those demonstrated by the DREAM trial,^14^ with other population-based studies demonstrating similar results.^32,33,34,35^ Although many reinterventions associated with EVAR have been shown to be minor, a 2018 systematic review and meta-analysis^36^ demonstrated perioperative complication rates of 3.8% and AAA-associated mortality of 1.8% associated with the treatment of type II endoleak alone. Considering that approximately 50% of type II endoleaks have been reported to resolve spontaneously, overtreatment may be increasing EVAR mortality.^37,38^ Unfortunately, large, contemporary, population-based studies, including our study, do not offer direct insight into whether changes in graft design have reduced reintervention and mortality. In this study, the assumption that modern endografts were being used for EVAR is not unreasonable, given that the commencement of EVAR funding in Ontario coincides with Health Canada approval of the first endograft and that all approved endografts in Canada are considered modern. However, no information on specific endograft makes and models is available in Ontario administrative data to verify these assumptions.

Another related explanation for the persistent loss of EVAR mortality benefit may be the more liberal use of EVAR against manufacturer instructions for use (IFU). Studies have found that despite relatively stable AAA repair rates, the use of EVAR has increased.^6,39,40,41^ Furthermore, a 2018 study^42^ showed that these rates have increased in populations known to have more challenging anatomy, including women and elderly patients. A 2018 study by Herman et al^43^ of EVARs from 2005 to 2014 demonstrated IFU violations in 43.8% of patients undergoing elective EVAR and that non-IFU EVARs were associated with higher risk of graft-related adverse events (HR, 1.8; 95% CI, 1.05-3.10). Furthermore, a 2017 international study^44^ of elective AAA repair outcomes during 9 years confirmed worse EVAR perioperative mortality in octogenarians (1.8% vs 0.7%; *P* < .001) and women (1.9% vs 0.9%; *P* < .001) during the course of the study, suggesting that the long-term benefits of EVAR may be undermined by too-forceful application. Unfortunately, Ontario administrative data do not allow assessment of adherence to IFU or difficult anatomy, as anatomical characteristics are not available within these data sets.

Additionally, contemporary studies of EVAR compared with OSR may not be demonstrating improvement in EVAR outcomes owing to concomitant improvements in OSR outcomes through time. The initial differences in perioperative mortality may be decreasing with the development of multidisciplinary care settings, including experienced cardiovascular intensive care units, that optimize OSR outcomes. This study, including data from all centers performing EVAR and OSR in Ontario, Canada, whether tertiary, academic, or otherwise, demonstrated 30-day survival for OSR comparable with the experienced centers involved in the seminal RCTs, largely well-resourced tertiary care centers with well-developed AAA care programs. In 2015, Schermerhorn et al^32^ demonstrated earlier merging of OSR and EVAR survival curves when they compared mortality prior to 2005 with mortality after 2005. These findings suggest that OSR outcomes may also be improving, thus masking improvements in EVAR outcomes.

Endovascular repair has demonstrated a persistent loss of long-term mortality benefit and high reintervention rates in both RCTs and population-based studies. Consequently, economic analyses have demonstrated EVAR to be cost ineffective, which has led the National Institute for Health and Care Excellence^45^ in the United Kingdom to recommend against EVAR in its draft of AAA management guidelines. However, the best evidence used to define these guidelines is limited to the seminal RCTs, which are themselves limited by older endografts and early knowledge regarding complications and management. Furthermore, EVAR offers potential benefits other than reduced mortality, despite higher reintervention rates. Many studies have uniformly demonstrated lower perioperative cardiopulmonary complications, shorter lengths of stay in hospitals, and higher rates of discharge home, among others.^2,3,4^ Furthermore, mortality does not reflect the considerable impact of myocardial infarction and stroke on quality of life among survivors of myocardial infarctions and strokes.^46,47,48^ Consequently, a case can be made for extension of EVAR to younger, healthier patients with longer life expectancy as opposed to reserving it for the most comorbid patient populations or not using it at all. However, the safety of this approach will require even longer follow-up for patients who undergo EVAR than is currently available, to our knowledge. These data will ascertain whether any long-term sequelae, such as cancer, exist secondary to EVAR surveillance and permit greater understanding of the appropriate decision-making approach regarding endoleak and complication management in the context of evolved endograft architecture.

### Limitations

The findings of this study should be interpreted considering several limitations. First, the use of diagnostic, procedure, and billings codes to identify patients, covariates, and outcomes is necessary with the use of administrative data. These codes are subject to coding error and can lack granularity. To mitigate these concerns, validated codes were used for the identification of patients and variables where possible, and surrogate markers were used for important covariates that were not coded. For example, chronic obstructive pulmonary disease was used as a marker of significant smoking history. Still, information regarding anatomical and technical detail, graft types, adherence to IFU, and indications for procedures, imaging, and clinical follow-up does not exist within Ontario administrative data. Next, the use of Ontario administrative data may limit the generalizability of our findings to jurisdictions with similar single-payer health care systems. However, our findings were similar to those of the major RCTs, in which payment model should not influence outcomes. Additionally, although they are firmly established for the conduct of observational studies, propensity score methods only guarantee balance of treatment groups on the measured confounders and do not guarantee absence of residual confounding. However, the congruity of our findings with those of long-term RCTs suggests well-specified propensity score models and findings likely to be free of residual confounding.

## Conclusions

This study found no statistically significant difference between outcomes after EVAR and OSR to repair AAA in long-term mortality during more than 13 years of follow-up. Similarly, there were no long-term differences in reintervention or secondary rupture between the AAA repair approaches. However, EVAR was associated with a higher long-term MACE-free survival rate. The long-term outcomes after EVAR do not appear to have improved more than OSR since the conduct of the seminal RCTs. The reasons for these findings will require further study, including consideration of specific graft makes and models, adherence to IFU, and types and reasons for reintervention.

## Notes

Supplement. **eTable 1.** Summary of Institute for Clinical Evaluative Sciences Databases Used **eTable 2.** Covariate and Outcome Definitions and Codes **eTable 3.** Survival and Cumulative Incidence Rates in Unmatched and Matched Cohorts **eTable 4.** Hazard Ratios From Cox Proportional Hazards Models for Matched and Inverse Probability of Treatment–Weighted Cohorts **eReferences.**
