# Supplementary material for: Comparison of Outcomes in Elective Endovascular Aortic Repair vs Open Surgical Repair of Abdominal Aortic Aneurysms
Source: JAMA Netw Open. 2019 Jul 10;2(7):e196578. doi: 10.1001/jamanetworkopen.2019.6578 (PMC6624804; doi:10.1001/jamanetworkopen.2019.6578)
Supplement: Supplement. — eTable 1. Summary of Institute for Clinical Evaluative Sciences Databases Used eTable 2. Covariate and Outcome Definitions and Codes eTable 3. Survival and Cumulative Incidence Rates in Unmatched and Matched Cohorts eTable 4. Hazard Ratios From Cox Proportional Hazards Models for Matched and Inverse Probability of Treatment–Weighted Cohorts eReferences. [file jamanetwopen-2-e196578-s001.pdf]

Supplementary Online Content

Salata K, Hussain MA, de Mestral C, et al. Comparison of outcomes in elective endovascular aortic repair vs open surgical repair of abdominal aortic aneurysms. *JAMA Netw Open*. 2019;2(7):e196578. doi:10.1001/jamanetworkopen.2019.6578

eTable 1. Summary of Institute for Clinical Evaluative Sciences Databases Used
eTable 2. Covariate and Outcome Definitions and Codes
eTable 3. Survival and Cumulative Incidence Rates in Unmatched and Matched Cohorts
eTable 4. Hazard Ratios From Cox Proportional Hazards Models for Matched and Inverse Probability of Treatment–Weighted Cohorts
eReferences.

This supplementary material has been provided by the authors to give readers additional information about their work.

© 2019 Salata K et al. *JAMA Network Open.*

eTable 1. Summary of Institute for Clinical Evaluative Sciences Databases Used

Database	Available from	Data Elements
CIHI-DAD	April 1988	Patient-level information including patient age, gender, location of residence, hospital of admission, up to 25 different diagnoses (ICD-9 codes prior to April 2002, ICD-10-CA after) and procedures (CCP codes prior to April 2002 and CCI afterward) (distinguished by most-responsible, pre-admission, and comorbid), treating physician, length of stay, disposition, and resource consumption.
CIHI-SDS	April 1991	Similar patient level information as CIHI-DAD as it pertains to same day surgery visits. Same coding systems
NACRS	July 2000	Similar patient level information as CIHI databases, however, capturing all ambulatory outpatient and emergency department visits. Codes used as above.
OHIP	July 1991	Fee for service and<br>shadow-billing claims for diagnostic, laboratory, and surgical services,<br>information on associated diagnoses for laboratory tests and surgical services, as well as the date of the services and physician identifiers.
RPDB	April 1990	Basic demographic information including age, sex, date of death and date last eligible for Ontario health card.
ODB	April 1990	Drug claims information for drugs covered by the Ministry of Health and Long Term Care. Contains prescription, pharmacy and prescribing physician information for all drug claims and drug services paid for by the Ministry of Health and Long Term Care of Ontario (patients 65 years of age and older, patients on special drug access and assistance programs, and patients participating in pharmacist medication reconciliations).
ODD	April 1991	ICES derived disease specific database that identifies all incident and prevalent diabetic patients within the province of Ontario.
HYPER	April 1991	ICES derived disease specific database that identifies all incident and prevalent hypertensive patients within the province of Ontario.
CHF	April 1991	ICES derived disease specific database that identifies all incident and prevalent congestive heart failure patients within the province of Ontario.
COPD	April 1991	ICES derived disease specific database that identifies all incident and prevalent COPD patients within the province of Ontario.
INST	April 1987	Ministry of Health database containing institutional information for all healthcare institutions funded by the Ministry of Health, including numbers of acute care beds, level of care and teaching vs. non-teaching status.

CCI=Canadian Classification of Health Interventions; CCP=Canadian Classification of Diagnostic, Therapeutic, and Surgical Procedures;

CHF=Ontario Congestive Heart Failure Database; CIHI-DAD=Canadian Institute for Health Information Discharge Abstract Database; CIHI-

© 2019 Salata K et al. *JAMA Network Open.*

SDS=Canadian Institute for Health Information Same Day Surgery Database; COPD=Ontario Chronic Obstructive Pulmonary Disease Database; HYPER=Ontario Hypertension Database; ICES=Institute for Clinical Evaluative Sciences; ICD-9= International Statistical Classification of Diseases, Injuries, and Causes of Death, Ninth Revision; ICD-10-CA=International Statistical Classification of Diseases, Injuries, and Causes of Death, Tenth Revision, Canada; INST=Institution Information System Database; NACRS=National Ambulatory Care Reporting System database; ODB=Ontario Drug Benefit Claims Database; ODD=Ontario Diabetes Database; OHIP=Ontario Health Insurance Plan (OHIP) database; RPDB=Registered Persons Database.

© 2019 Salata K et al. *JAMA Network Open.*

eTable 2. Covariate and Outcome Definitions and Codes

Covariate/Outcomes	Definitions/Codes
**Demographics**	
Age	Abstracted directly from CIHI-DAD/SDS, NACRS, or RPDB.
Sex	Abstracted directly from CIHI-DAD/SDS, NACRS, or RPDB.
Income quintile	Income quintile was determined by linking each patient's home postal code to census data from Statistics Canada to establish neighborhood income quintiles adjusted for household size as well as housing cost.
Rurality	Patient rurality was measured from the Registered Persons Database (RPDB) and defined as residence in an area with population <10,000.
**Healthcare utilization**	
# physician visits within 1 year of index procedure	Direct counts of non-acute physician visits within 1 year of index procedure, based on outpatient OHIP billing claims. Maximum one visit/claim per day.
# emergency department visits within 3 years of index procedure	Direct counts of emergency department visits within 1 year of index procedure, abstracted from NACRS. Maximum one visit per day.
# hospital admissions within 3 years of index procedure	Direct counts of acute care hospital admissions abstracted from CIHI-DAD. Maximum one visit per day.
**Hospital and procedure characteristics**	
Hospital type	Abstracted directly from the INST database using the teaching hospital flag.
Year of procedure	Abstracted directly from CIHI-DAD/SDS or OHIP.
AAA repair volume [1]	Calculated separately for each repair approach and coded as high or low according to the minimum recommended volumes defined by the Society for Vascular Surgery for proficiency in AAA repair (minimum 10 per year).
**Comorbidities**	
HTN[2]	Diagnosis date in Ontario Hypertension Database that precedes index date.
DM[3]	Diagnosis date in Ontario Diabetes Database that precedes index date.
CHF[4]	Diagnosis date in Ontario Congestive Heart Failure Database that precedes index date.

© 2019 Salata K et al. *JAMA Network Open.*

CAD[5-8]	**ICD-9/CCP:** 410.^, 411.^, 412, 413.^(except 413.1, 414.^(except 414.1), 429.2, 429.5, 429.6, 429.7; 48.1^, 48.2^, 48.3^<br><br>**ICD-10-CA/CCI:** I20.^, I21.^, I22.^, I.23.^, I.24.^, I25.^, R93.1, T82.2, Z95.5, Z95.8, Z95.9; 1.IJ.50.^, 1.IJ.57.^, 1.IJ.76.^<br><br>**OHIP:**E646, E651, E652, E654, E655, G262, G298, R741, R742, R743, Z434
MI[5-7]	**ICD-9/CCP:** 410.^<br><br>**ICD-10-CA/CCI:** I21.^, I22.^
CVD[5, 9, 10]	**ICD-9/CCP:** 433.^0, 434.^0<br><br>**ICD-10-CA/CCI:** I65.^, I.66^
Stroke/TIA[5, 9, 10]	**ICD-9/CCP:** 362.3^, 433.^1, 434.^1, 435.^, 436<br><br>**ICD-10-CA/CCI:** I63.^, I64.^, G45.^ (except G45.4), H34.1
PAD[11]	**ICD-9/CCP:** 440.2^, 440.9, 443.9<br><br>**ICD-10-CA/CCI:** I70.9, I70.2, I73.9; I74.3, I74.4; *1.WK.93.^, *1.WL.93.^, *1.WM.93.^, *1.WN.93.^<br>*(exclude if accompanied by ICD-10 code C40.^, D16.^, D48.0, D48.1, D48.2, Q65.^-Q79.^, S70.^ – S99.^, T20.^ – T32.^), 3.KG.10.^, 3.KG.20.^, 3.KG.30.^, 3.KG.40.^
COPD[12]	Diagnosis date in Ontario COPD Database that precedes index date
CKD[13]	**ICD-9/CCP:** 403.^, 404.^, 582.^, 583.0 - 583.7, 585.^, 586, 588.^, V42.0, V45.1^, V56.^<br><br>**ICD-10-CA/CCI:** N03.2-N03.7, N05.2-N05.7, N18.^, N19.^, N25.0, Z49.^, Z94.0, Z99.2
Coronary revascularization[14]	**ICD-9/CCP:** 48.02, 48.03, 48.1^<br><br>**ICD-10-CA/CCI:** 1.IJ.50.^, 1.IJ.54.^, 1.IJ.57.GQ.^, 1.IJ.76.^
Peripheral revascularization[6, 15]	**ICD-9/CCP:** 51.29<br><br>**ICD-10-CA/CCI:** 1.KG.76.^<br><br>**OHIP:***J021, *J022, *J025, *J027, *J056, *J058<br>*(except if associated with OHIP codes 435, 436, 437, 584, 585, 593.8, 403)
Major amputation	**ICD-9/CCP:** 96.1^, 96.2<br><br>**ICD-10-CA/CCI:** 1.VC.93.^, 1.VG.93, 1.VQ.93

© 2019 Salata K et al. *JAMA Network Open.*

Carotid revascularization[5]	**ICD-9/CCP:** 50.12<br><br>**ICD-10-CA/CCI:** 1.JE.57.LA-^, 1.JE.50.^<br><br>**OHIP:**R792
Charlson Score[13]	Calculated using the ICES CHARLSON macro, using the codes and weighting in the indicated source paper.
Cataract surgery	**ICD-9/CCP:** 27.2, 27.4, 27.41, 27.49, 27.5^, 27.6^, 27.7^<br><br>**ICD-10-CA/CCI:** 1.CL.53.^, 1.CL.54.^<br>1.CL.55.^ (except 1.CL.55.LA-FE),<br>1.CL.56.LA.^, 1.CL.59.^,<br>1.CL.87.LA.^, 1.CL.89.^
Malignancy	**ICD-9/CCP:** 150.^, 154.^, 155.^, 157.^, 162.^, 174.^, 175.^, 185.^, 203.^, 204.^, 205.^, 206.^, 207.^, 208.^<br><br>**ICD-10-CA/CCI:** C15.^, C18.^, C19.^, C20.^, C22.^, C25.^, C34.^, C50.^, C56.^, C61.^, C82.^, C83.^, C85.^, C91.^, C92.^, C93.^, C94.^, C95.^, D00.^, D05.^<br><br>**OHIP:**203, 204, 205, 206, 207, 208, 150, 154, 155, 157, 162, 174, 175, 183, 185
**Medications**	
Statins	All drugs from ODB with subclass name “ANTI-LIPEMIC: STATINS”.
Beta-blockers	All drugs from ODB with subclass name “BETA-BLOCKERS”, “BETA-BLOCKERS COMBINATION”, “BETA-BLOCKING AGENTS”.
ACEi/ARB	All drugs from ODB with subclass name “ACE INHIBITORS”, “ACE INHIBITORS COMBINATION”, “ANGIOTENSIN II ANTAGONIST”, “ANGIOTENSIN II COMBINATION”.
Anti-platelet agents	All drugs from ODB with subclass name “PLATELET-REDUCING AGENT”, FIBRINOGEN-PLATELET BINDING INHIBITORS”, “PLATELET AGGREGATION INHIBITORS”, “ADENOSIDE DIPHOSPHATE INHIBITORS”.
Anti-coagulants	All drugs from ODB with subclass name “HEPARINS”, “VITAMIN K ANTAGONISTS”, “ANTICOAGULANTS”, “ANTICOAGULANTS MISCELLANEOUS”, “SYNTHETIC ANTITHROMBOTIC AGENTS”, “LOW MOLECULAR WEIGHT HEPARINS (LMWH)”.
Prednisone	All drugs from ODB with drug name “PREDNISONE”
Anti-diabetics	All drugs from ODB with subclass name “ORAL ANTI-GLYCEMICS”
Insulins	All drugs from ODB with subclass name “INSULINS”

© 2019 Salata K et al. *JAMA Network Open.*

Fluoroquinolone	All drugs from ODB with subclass name "FLUOROQUINOLONES"
**Primary outcomes**	
Death	Death from CIHI-DAD for inpatient death, and from RPDB for outpatient.
**Secondary outcomes**	
MACE	First of Death, MI (**ICD-10-CA/CCI:** I21.^, I22.^) OR Stroke (**ICD-10-CA/CCI:** I60.^, I61.^, I62.^, I63.^, I64.^, H34.1 (excluding I63.6))
Re-intervention	**ICD-10-CA/CCI:** 1.ID.55.LA-NR-^ (except 1.ID.55.LA-NR), 1.ID.57.^ (except 1.ID.57.LA-AG, 1.ID.57.LA-AG-^, 1.ID.57.GQ-AG, and 1.ID.57.GQ-AG-^), 1.ID.80.GQ-NR-N, 1.ID.86-ME-^, 1.ID.87.^, 1.IS.51.LA, 1.IS.57.LA-GX-^, 1.IS.80.^, 1.IS.87.^, 1.JM.76.MI-XX-N, 1.KA.55.LA-NR-^,1.KA.57.^ (except 1.KA.57.LA-AG-^ and 1.KA.57.GQ-AG-^), 1.KA.76.^, 1.KA.80.^, 1.KA.82.^, 1.KE.35.HH-C1, 1.KE.35.HH-1C, 1.KE.50.GQ-^(except 1.KE.50.GQ-BF), 1.KE.50.LA-^, 1.KE.51.LA, 1.KE.51.GQ-^(except 1.KE.51.GQ, and 1.KE.51.GQ-M0), 1.KE.55.LA-NR, 1.KE.57.LA-^(except 1.KE.57.LA-AG-^ and 1.KE.57.LA-OB-^), 1.KE.57.GQ-AA (except 1.KE.57.GQ-AG-^ and 1.KE.57.GQ-OB-^), 1.KE.87.^, 1.KG.35.HH-C1, 1.KG.35.HH-1C, 1.KG.76.MI-XX-N, 1.KG.80.LA-^, 1.KQ.80.^, 1.KR.78.^, 1.KR.80.^ (except 1.KR.80.LA-FG), 1.KT.50.^, 1.KT.51.^, 1.KT.55.^, 1.KT.57.LA-^(except 1.KT.57.LA-AG-^ and 1.KT.57.LA-OB-^), 1.KT.57.GQ-AA (except 1.KT.57.GQ-AG-^ and 1.KT.57.GQ-OB-^), 1.KT.80.^, 1.KT.82.^, 1.KT.87.^, 1.NK.87.^, 1.NM.87, 1.NM.89, 1.NP.72.^, 1.OT.72.^, 1.SY.80.^,*1.VC.93.^, *1.VG.93, *1.VQ.93, *1.WE.93.^, *1.WI.93.^, *1.WJ.93.^, *1.WK.93.^, *1.WL.93.^, *1.WM.93.^, *1.WN.93.^, *1.KG.76.^3.ID.12.^, 3.IS.12.^, 3.KC.12.^, 3.KT.12.^, 3.KG.12.^<br>*(exclude if associated with any of the following primary diagnosis codes C40.^, D16.^, D48.0, D48.1, D48.2, Q65.^-Q79.^, S70.^–S99.^, T20.^–T32.^)<br><br>**OHIP:** E793, *J021, *J022, *J026, *J027, *J040, *J056, *J058, R624, R625, R626, R802, R808, R813, R814, R817, R820, R855, R858, R859, R864, R867, R873, R875, R877, R879, R932, R933, S312, S329, S330, S332, S340, S342, S343, S344, X174, X175<br>*(exclude if associated with the following OHIP diagnosis codes: 435, 436, 437, 584, 585, 403)
Secondary rupture	**ICD-10-CA/CCI:** I71.3<br><br>**OHIP:** E627

^ indicates inclusion of root code and all sub-codes unless otherwise specified.

CAD=Coronary Artery Disease; CCP= Canadian Classification of Diagnostic, Therapeutic and Surgical Procedures; CCI= Canadian Classification of Health Intervention; CHF=Congestive Heart Failure; CIHI-DAD=Canadian Institute for Health Information Discharge Abstract Database; CIHI-SDS=Canadian Institute for Health Information Same Day Surgery Database; CKD=Chronic Kidney Disease; COPD=Chronic Obstructive Pulmonary Disease; ICD-9=International Statistical Classification of Diseases and Related Health Problems 9th Revision; ICD-10-CA=International

© 2019 Salata K et al. *JAMA Network Open.*

Statistical Classification of Diseases and Related Health Problems 10th Revision Canadian Version; ICES=Institute for Clinical Evaluative Sciences; MACE=Major adverse cardiovascular events; MI=Myocardial Infarction; NACRS=National Ambulatory Care Reporting System database; OHIP=Ontario Health Insurance Plan; PAD=Peripheral Arterial Disease; RPDB=Registered Persons Database; TIA=transient ischemic attack.

© 2019 Salata K et al. *JAMA Network Open.*

eTable 3. Survival and Cumulative Incidence Rates in Unmatched and Matched Cohorts

UNMATCHED						MATCHED					
**Outcome**	**EVAR (N=6,100)**			**OSR (N=11,583)**			**EVAR (N=4,010)**		**OSR (N=4,010)**		
**Mortality**	**Survival**	**95% CI**		**Survival**	**95% CI**		**Survival**	**95% CI**		**Survival**	**95% CI**	

Outcome	EVAR (N=6,100) Survival	EVAR 95% CI Lower	EVAR 95% CI Upper	OSR (N=11,583) Survival	OSR 95% CI Lower	OSR 95% CI Upper	Matched EVAR (N=4,010) Survival	95% CI Lower	95% CI Upper	Matched OSR (N=4,010) Survival	95% CI Lower	95% CI Upper
**Mortality**												
30-day	98.5	98.2	98.8	96.7	96.4	97.0	98.9	98.5	99.1	96.1	95.4	96.6
1-year	92.8	92.2	93.5	92.5	92.0	92.9	94.0	93.3	94.7	91.0	90.1	91.9
2-year	86.9	86.0	87.7	88.8	88.2	89.4	89.1	88.1	90.1	87.2	86.1	88.2
3-year	80.8	79.7	81.8	84.6	83.9	85.3	84.2	83.0	85.4	82.2	80.9	83.4
4-year	73.9	72.7	75.1	80.0	79.3	80.8	78.0	76.5	79.3	77.5	76.1	78.9
5-year	67.7	66.3	69.1	74.8	74.0	75.7	72.5	70.9	74.0	71.4	69.8	73.0
6-year	61.9	60.3	63.4	69.8	68.9	70.7	67.0	65.2	68.7	66.2	64.4	67.9
7-year	55.0	53.2	56.8	64.4	63.4	65.4	60.3	58.2	62.3	60.1	58.0	62.0
8-year	48.8	46.7	50.8	59.4	58.4	60.5	54.1	51.7	56.4	54.8	52.5	57.1
9-year	44.5	42.2	46.9	54.1	53.0	55.1	49.5	46.8	52.1	49.4	46.7	52.0
10-year	38.3	35.2	41.5	48.7	47.5	49.8	42.5	39.0	46.0	44.2	40.8	47.4
Max.	25.1	17.0	34.0	30.2	28.1	32.4	41.5	37.7	45.2	26.9	15.7	39.5
**MACE**												
30-day	96.3	95.8	96.7	90.9	90.3	91.4	97.0	96.4	97.5	90.8	89.9	91.7
1-year	89.2	88.4	90.0	86.1	85.5	86.7	90.8	89.9	91.7	85.2	84.1	86.3
2-year	82.4	81.4	83.3	81.7	81.0	82.4	85.2	84.1	86.3	80.6	79.3	81.8
3-year	75.7	74.6	76.8	77.1	76.3	77.9	79.4	78.1	80.7	75.2	73.8	76.5
4-year	68.6	67.3	69.8	72.1	71.2	72.9	72.9	71.4	74.4	69.9	68.3	71.3
5-year	62.1	60.6	63.5	66.7	65.8	67.6	66.9	65.2	68.6	63.7	62.0	65.3
6-year	55.8	54.2	57.4	61.6	60.7	62.6	60.7	58.9	62.6	58.4	56.6	60.2
7-year	49.3	47.4	51.0	56.2	55.2	57.2	54.0	51.9	56.1	52.3	50.2	54.3
8-year	43.5	41.4	45.5	51.3	50.3	52.4	48.2	45.9	50.5	47.1	44.8	49.3
9-year	38.9	36.6	41.2	46.2	45.2	47.3	43.4	40.8	46.0	42.2	39.6	44.8
10-year	33.2	30.1	36.2	41.3	40.2	42.4	36.9	33.5	40.4	36.9	33.7	40.2
Max.	29.2	24.2	34.5	24.7	22.7	26.7	32.6	26.9	38.4	14.1	4.0	30.4

© 2019 Salata K et al. *JAMA Network Open.*

Re-intervention	Cumulative Incidence	95% CI		Cumulative Incidence	95% CI		Cumulative Incidence	95% CI		Cumulative Incidence	95% CI	
30-day	13.7	12.8	14.5	14.4	13.8	15.1	12.4	11.4	13.5	15.0	13.9	16.1
1-year	22.6	21.5	23.6	20.8	20.1	21.6	21.4	20.2	22.7	21.0	19.8	22.3
2-year	28.6	27.4	29.7	27.3	26.5	28.1	27.2	25.9	28.6	27.0	25.7	28.4
3-year	33.3	32.1	34.5	31.9	31.0	32.8	32.2	30.7	33.7	31.5	30.1	33.0
4-year	37.1	35.8	38.4	35.5	34.6	36.4	36.2	34.6	37.7	35.1	33.5	36.6
5-year	40.3	38.9	41.6	38.6	37.7	39.5	39.7	38.1	41.4	38.0	36.4	39.6
6-year	43.4	41.9	44.8	41.2	40.3	42.2	42.9	41.2	44.6	40.2	38.5	41.9
7-year	46.2	44.6	47.7	43.6	42.6	44.6	45.9	44.1	47.8	42.2	40.4	44.0
8-year	48.0	46.3	49.6	45.6	44.6	46.6	47.8	45.8	49.8	44.0	42.1	45.9
9-year	48.9	47.1	50.6	46.9	45.9	47.9	48.8	46.7	50.8	44.7	42.7	46.7
10-year	50.4	48.3	52.4	48.4	47.4	49.4	50.1	47.8	52.4	46.3	44.0	48.6
Max.	51.8	49.2	54.4	51.4	50.3	52.6	51.8	48.8	54.7	49.6	42.5	56.3
**Secondary Rupture**												
30-day	0.5	0.3	0.7	0.7	0.5	0.8	0.5	0.3	0.8	0.5	0.4	0.8
1-year	0.5	0.4	0.8	0.8	0.6	0.9	0.5	0.3	0.8	0.6	0.4	0.9
2-year	0.7	0.5	1.0	0.8	0.7	1.0	0.7	0.4	0.9	0.7	0.5	1.0
3-year	0.8	0.6	1.1	0.9	0.7	1.0	0.7	0.5	1.0	0.7	0.5	1.0
4-year	0.9	0.7	1.2	0.9	0.7	1.1	0.8	0.6	1.2	0.7	0.5	1.0
5-year	1.0	0.7	1.3	0.9	0.7	1.1	0.9	0.6	1.2	0.8	0.5	1.1
6-year	1.0	0.8	1.3	0.9	0.8	1.1	0.9	0.6	1.3	0.8	0.6	1.2
7-year	1.2	0.9	1.5	1.0	0.8	1.2	1.0	0.7	1.5	0.8	0.6	1.2
8-year	1.2	0.9	1.5	1.0	0.8	1.2	1.0	0.7	1.5	0.8	0.6	1.2
9-year	1.2	0.9	1.5	1.0	0.8	1.2	1.0	0.7	1.5	0.8	0.6	1.2
10-year	1.6	1.0	2.4	1.1	0.9	1.3	1.5	0.9	2.5	0.8	0.6	1.2
Max.	1.6	1.0	2.4	1.2	1.0	1.4	1.5	0.9	2.5	0.8	0.6	1.2

All values presented as %.

© 2019 Salata K et al. *JAMA Network Open.*

CI=confidence interval; EVAR=endovascular aortic repair; MACE=major adverse cardiovascular events; Max.=maximum follow-up; OSR=open surgical repair.

© 2019 Salata K et al. *JAMA Network Open*.

eTable 4: Hazard Ratios From Cox Proportional Hazards Models for Matched and Inverse Probability of Treatment–Weighted Cohorts

	MATCHED				IPTW			
**Outcome**	**HR**	**95% CI**		**p**	**HR**	**95% CI**		**p**
**Mortality**								
0 – 45 days	0.29	0.22	0.40	<0.001	0.30	0.23	0.39	<0.001
>45 – 90 days	0.75	0.45	1.23	0.25	1.16	0.73	1.86	0.53
>90 days – 6 months	0.93	0.62	1.38	0.71	0.94	0.60	1.45	0.77
>6 months – 1 year	1.17	0.88	1.54	0.27	1.20	0.86	1.68	0.29
>1 – 4 years	1.16	1.02	1.32	0.02	1.19	1.04	1.36	0.01
>4 years	1.01	0.89	1.15	0.91	1.02	0.90	1.16	0.71
**MACE**								
0 – 45 days	0.32	0.27	0.39	<0.001	0.34	0.29	0.41	<0.001
>45 days – 1 year	1.12	0.93	1.35	0.25	1.16	0.93	1.44	0.18
>1 – 7 years	1.07	0.98	1.18	0.14	1.10	1.00	1.21	0.05
>7 years	0.99	0.77	1.28	0.95	0.96	0.77	1.20	0.71
**Re-intervention**								
0 – 30 days	0.74	0.66	0.83	<0.001	0.78	0.70	0.88	<0.001
>30 days – 6 months	1.36	1.12	1.65	0.002	1.53	1.22	1.90	<0.001
>6 months – 2 years	1.09	1.00	1.19	0.06	1.09	0.95	1.24	0.22
>2 years	1.11	1.01	1.22	0.03	1.15	1.05	1.27	0.003
**Secondary Rupture**								
0 – 1.5 years	0.92	0.53	1.59	0.75	1.00	0.54	1.86	1.00
>1.5 – 4 years	2.29	0.59	8.85	0.23	1.67	0.35	7.89	0.52
>4 years	3.01	0.61	14.91	0.18	2.12	0.71	6.32	0.18

All hazard ratios presented as endovascular aortic repair relative to open surgical repair.

IPTW=inverse probability of treatment weighted; HR=hazard ratio; MACE=major adverse cardiovascular events; CI=confidence interval.

© 2019 Salata K et al. *JAMA Network Open.*

eReferences

1. Chaikof EL, Dalman RL, Eskandari MK, Jackson BM, Lee WA, Mansour MA, et al. The Society for Vascular Surgery practice guidelines on the care of patients with an abdominal aortic aneurysm. *J Vasc Surg*. 2018;67:2-77 e2.

2. Tu K, Campbell NR, Chen ZL, Cauch-Dudek KJ and McAlister FA. Accuracy of administrative databases in identifying patients with hypertension. *Open Med*. 2007;1:e18-26.

3. Hux JE, Ivis F, Flintoft V and Bica A. Diabetes in Ontario: determination of prevalence and incidence using a validated administrative data algorithm. *Diabetes Care*. 2002;25:512-6.

4. Schultz SE, Rothwell DM, Chen Z and Tu K. Identifying cases of congestive heart failure from administrative data: a validation study using primary care patient records. *Chronic diseases and injuries in Canada*. 2013;33:160-6.

5. Hussain MA, Mamdani M, Saposnik G, Tu JV, Turkel-Parrella D, Spears J, et al. Validation of Carotid Artery Revascularization Coding in Ontario Health Administrative Databases. *Clin Invest Med*. 2016;39:E73-8.

6. Juurlink D PC, Croxford R, Chong A, Austin P, Tu J, Laupacis A. Canadian Institute for Health Information Discharge Abstract Database: A Validation Study. 2006.

7. Austin PC, Daly PA and Tu JV. A multicenter study of the coding accuracy of hospital discharge administrative data for patients admitted to cardiac care units in Ontario. *Am Heart J*. 2002;144:290-6.

8. Tu K, Mitiku T, Lee DS, Guo H and Tu JV. Validation of physician billing and hospitalization data to identify patients with ischemic heart disease using data from the Electronic Medical Record Administrative data Linked Database (EMRALD). *Can J Cardiol*. 2010;26:e225-8.

9. Hall R, Mondor L, Porter J, Fang J and Kapral MK. Accuracy of Administrative Data for the Coding of Acute Stroke and TIAs. *Can J Neurol Sci*. 2016;43:765-773.

© 2019 Salata K et al. *JAMA Network Open.*

10. Kokotailo RA and Hill MD. Coding of stroke and stroke risk factors using international classification of diseases, revisions 9 and 10. *Stroke*. 2005;36:1776-81.

11. Tu JV, Chu A, Donovan LR, Ko DT, Booth GL, Tu K, et al. The Cardiovascular Health in Ambulatory Care Research Team (CANHEART): using big data to measure and improve cardiovascular health and healthcare services. *Circ Cardiovasc Qual Outcomes*. 2015;8:204-12.

12. Gershon AS, Wang C, Guan J, Vasilevska-Ristovska J, Cicutto L and To T. Identifying individuals with physcian diagnosed COPD in health administrative databases. *COPD*. 2009;6:388-94.

13. Quan H, Sundararajan V, Halfon P, Fong A, Burnand B, Luthi JC, et al. Coding algorithms for defining comorbidities in ICD-9-CM and ICD-10 administrative data. *Med Care*. 2005;43:1130-9.

14. Lee DS, Stitt A, Wang X, Yu JS, Gurevich Y, Kingsbury KJ, et al. Administrative hospitalization database validation of cardiac procedure codes. *Med Care*. 2013;51:e22-6.

15. Al-Omran M, Tu JV, Johnston KW, Mamdani MM and Kucey DS. Outcome of revascularization procedures for peripheral arterial occlusive disease in Ontario between 1991 and 1998: a population-based study. *J Vasc Surg*. 2003;38:279-88.

© 2019 Salata K et al. *JAMA Network Open.*
